# The K^+^ transporter NPF7.3/NRT1.5 and the proton pump AHA2 contribute to K^+^ transport in *Arabidopsis thaliana* under K^+^ and NO_3_^-^ deficiency

**DOI:** 10.3389/fpls.2023.1287843

**Published:** 2023-11-10

**Authors:** Florencia Sena, Reinhard Kunze

**Affiliations:** ^1^ Institute of Biology/Applied Genetics, Dahlem Centre of Plant Sciences, Freie Universität Berlin, Berlin, Germany; ^2^ Laboratory of Apicomplexan Biology, Institut Pasteur Montevideo, Montevideo, Uruguay; ^3^ Laboratorio de Bioquímica, Facultad de Agronomía, Universidad de la República, Montevideo, Uruguay

**Keywords:** *Arabidopsis thaliana*, nitrate deficiency, potassium deficiency, potassium transport, protein-protein interaction, plasma membrane, H^+^ -ATPase

## Abstract

Nitrate (NO_3_^-^) and potassium (K^+^) are distributed in plants *via* short and long-distance transport. These two pathways jointly regulate NO_3_^-^ and K^+^ levels in all higher plants. The *Arabidopsis thaliana* transporter NPF7.3/NRT1.5 is responsible for loading NO_3_^-^ and K^+^ from root pericycle cells into the xylem vessels, facilitating the long-distance transport of NO_3_^-^ and K^+^ to shoots. In this study, we demonstrate a protein-protein interaction of NPF7.3/NRT1.5 with the proton pump AHA2 in the plasma membrane by split ubiquitin and bimolecular complementation assays, and we show that a conserved glycine residue in a transmembrane domain of NPF7.3/NRT1.5 is crucial for the interaction. We demonstrate that AHA2 together with NRT1.5 affects the K^+^ level in shoots, modulates the root architecture, and alters extracellular pH and the plasma membrane potential. We hypothesize that NRT1.5 and AHA2 interaction plays a role in maintaining the pH gradient and membrane potential across the root pericycle cell plasma membrane during K^+^ and/or NO_3_^-^ transport.

## 1 Introduction

Potassium (K) and nitrogen (N) are essential macronutrients of higher plants. Shortage or excess of these nutrients in soils limit growth and agricultural yield. While potassium is available to plants only as the cation K^+^, N occurs in soils as inorganic and organic molecules, where nitrate (NO_3_^-^) is the prevalent form (Xu et al., 2012). For nutrient uptake by the roots and distribution throughout the sporophyte *via* xylem and phloem, plants have evolved a large number of membrane transporters and channels with different specificities and affinity ranges. In *Arabidopsis thaliana* NO_3_^-^ transporters and channels are encoded by four gene families (reviewed by Krapp et al., 2014): (1) the *NRT1/PTR* (nitrate transporter 1/peptide transporter) family, renamed as *NPF* family, (2) the *NRT2/NNP* (nitrate transporter 2/nitrate-nitrite porter) family, (3) the *CLC* (chloride channel) family, and (4) the *SLAC1/SLAHs* (slow-type anion channel-associated 1/homologues) family. K^+^ transport is accomplished by the three channel families *Shaker*, *TPK* (tandem-pore K^+^) and *Kir-like* (K^+^ inward rectifier-like) (Very and Sentenac, 2003), and the three transporter families *KUP/HAK/KT*, *HKT* (high-affinity K^+^ transporters) and *CPA* (cation-proton antiporters) (Gierth and Maser, 2007; Chanroj et al., 2012).

The intersection between K and N physiology in plant cells is not yet fully understood. K^+^ plays an essential role as a counter ion of NO_3_^-^, facilitating the uptake, translocation, and distribution of these ions between roots and shoots (reviewed in Ródenas et al., 2017 ). In contrast, as plant K^+^ transporters and channels can also transport ammonium ions (NH_4_^+^), K^+^ and NH_4_^+^ compete for uptake and translocation (Ten Hoopen et al., 2010). In addition, the expression of several NRT1 members (i.e. NRT1.1, NRT1.4, NRT1.5, NRT1.8) is influenced directly or indirectly by K^+^ nutrition (Drechsler et al., 2015; Li et al., 2017; Fang et al., 2020; Morales de Los Ríos et al., 2021), indicating regulatory interactions between the K^+^ and NO_3_^-^ transport pathways.

The nitrate transporter NPF7.3/NRT1.5 (subsequently termed NRT1.5) was initially characterized as a proton-coupled, low-affinity nitrate transporter involved in long-distance NO_3_^-^ transport in *A. thaliana* (Lin et al., 2008). It is expressed in root pericycle cells close to the xylem vessels and localizes to the plasma membrane (PM), suggesting its involvement in xylem loading of nitrate. Subsequently, the expression of *NRT1.5* was found to be dependent both on nitrate and potassium availability. Under low nitrate nutrition, in *nrt1.5* mutants the root-to-shoot potassium transport is reduced, suggesting a regulatory loop at the level of xylem transport that maintains the balance between nitrate and potassium (Drechsler et al., 2015). A direct link of NRT1.5 to NO_3_^-^ and K^+^ homeostasis is supported by several observations. (1) Both NRT1.5 and the K^+^ transporter SKOR contribute to K^+^ translocation from root to shoot, with SKOR activity dominating under high NO_3_^-^ and low K^+^ supply, and NRT1.5 under low NO_3_^-^ availability (Drechsler et al., 2015). (2) NRT1.5 was reported to perceive nitrate starvation-derived signals to prevent leaf senescence by facilitating foliar potassium accumulation (Meng et al., 2016). This might explain why *NRT1.5* is highly upregulated during leaf senescence (van der Graaff et al., 2006). (3) Lateral root formation depends on *NRT1.5* under K^+^ deprivation and low supply of NO_3_^-^ (Zheng et al., 2016). (4) NRT1.5 functions as a proton-coupled H^+^/K^+^ antiporter that facilitates K^+^ release from the root parenchymatous pericycle cells and K^+^ loading into the xylem (Li et al., 2017). (5) Previous studies have shown that *NRT1.5* expression is modulated by changes in K^+^ and NO_3_^-^ levels (Lin et al., 2008; Chen et al., 2012; Drechsler et al., 2015; Meng et al., 2016; Li et al., 2017). The transcription factor MYB59 positively regulates *NRT1.5* expression in roots under low K^+^-stress and thus has a key role in maintaining K^+^/NO_3_^–^ homeostasis between roots and shoots (Du et al., 2019).

Besides transporting nitrate and potassium, NRT1.5 was shown to function as an indole-3-butyric acid (IBA) transporter in *A. thaliana* root tips, where IBA is converted to the auxin indole-3-acetic acid that regulates root gravitropism (Watanabe et al., 2020). NRT1.5 was also reported to be an important component in the regulation and modulation of phosphate deficiency responses (Cui et al., 2019).

NRT1.5 is energized by the H^+^-gradient across the PM, which is established by H^+^-ATPases that extrude protons from the cytoplasm by ATP hydrolysis. In *A. thaliana* 11 different members of PM H^+^-ATPases are encoded by the *AHA* (Arabidopsis H^+^-ATPase) multigene family, with AHA1 and AHA2 being the two major isoforms (Falhof et al., 2016). Proton pumps are known to contribute to nutrient assimilation during root uptake and short and long-distance transport (Sze and Chanroj, 2018; Feng et al., 2020).

The complex regulation of NRT1.5, its dependence on an electrochemical H^+^-gradient across the PM, and its ability to transport diverse substrates prompted us to investigate whether NRT1.5 interacts with other PM proteins that might directly or indirectly influence its activity and regulation. In a split-ubiquitin-screen, we discovered the proton pump AHA2 to interact with NRT1.5 and confirmed the interaction by BiFC analysis in tobacco plants. The physiological responses of Arabidopsis wild-type plants and *nrt1.5* and *aha2* mutant plants under different NO_3_^-^ and K^+^ regimes corroborated an interplay between NRT1.5 and AHA2 to control plant K^+^ and H^+^ homeostasis.

## 2 Materials and methods

### 2.1 Plant material and growth conditions

*Arabidopsis thaliana* T-DNA insertion lines were obtained from the Arabidopsis Biological Resource Center. The knockout *nrt1.5* (GABI_347B03) and *aha2* (GABI_219D04) mutant lines are in the Col-0 background. The double mutant *nrt1.5/aha2* was selected from the F2 progeny of the *nrt1.5* × *aha2* cross. The absence of full-length *NRT1.5* and *AHA2* transcripts in the homozygous mutants was verified by RT-PCR (all oligonucleotides used in this work are listed in **Supplementary Table 1**).

For growth assays on plates, Arabidopsis seeds were surface sterilized with 70% EtOH for 2 min, followed by 10% NaClO and 1% SDS for 3 min, and then washed 3 times for 3 min in sterile deionized water. Seeds were placed on 0.5 × MS plates containing 1% sucrose and 0.3% Gelrite and stratified in darkness for 2 days at 4°C, followed by pre-germination under long-day conditions (16 h/8 h light/dark, 22°C, light intensity 120 µmol m^-2^ s^-1^, and relative humidity 40%) for 5 days. Modified 0.5 × MS medium lacking potassium and nitrogen (“MS-base”: 1.5 mM CaCl_2_, 1 mM MgSO_4_, 0.5 mM NaH_2_PO_4_, 0.05 mM H_3_BO_3_, 0.05 mM MnSO_4_, 0.05 mM FeNa-EDTA, 15 µM ZnSO_4_, 2.5 µM KI, 0.5 µM Na_2_MoO_4,_ 0.05 µM CuSO_4_, 0.05 µM CoCl_2_, 0.5% MS vitamins, 0.5 g/l MES, 1% sucrose, pH 5.5) was supplemented with KCl and Ca(NO_3_)_2_ to different concentrations (**Supplementary Table 2**).

For growth assays on soil, plants were pre-germinated on a 1:1 mixture of commercial type P soil and unfertilized type 0 soil (Einheitserdewerk Uetersen) under long-day growth conditions (16 h/8 h light/dark, 21°C/18°C day/night, light intensity 120 µmol m^-2^ s^-1^, relative humidity 55% to 70%). After 5 days, the seedlings were transferred to either the same soil as control or to unfertilized soil supplemented with different nutrient solutions (**Supplementary Table 2**).

At the end of each fertilization experiment the frequency of chlorotic leaves (chlorophyll degradation/cell death) was determined for each genotype in 20 individual plants.

### 2.2 DNA isolation

Frozen *Arabidopsis thaliana* tissue was ground in liquid N_2_, mixed, and homogenized together with 2 metal balls by a mixer mill (Retsch MM 400). Genomic DNA from *A. thaliana* plants was extracted from frozen plant material according to Chen and Ronald (1999) with modification. 600 µl of 2% CTAB buffer (100 mM Tris-Cl pH 8, 1.4 M NaCl, 20 mM EDTA, and 2% CTAB w/v) was added to the ground material and mixed by vortexing, followed by incubation at 65°C for 20 min. The tubes were inverted every 5 min and were centrifuged at room temperature for 10 min at 13000 g. The supernatant was transferred to a fresh tube with one volume chloroform:isoamyl alcohol (24:1) and mixed well by inverting, followed by centrifuging at 13000 g for 5 min. The upper phase was transferred to a fresh tube and mixed with one volume isopropanol, followed by incubating at 4°C for 10 min.

Precipitated DNA was obtained by centrifuging at 13000 g for 10 min. The pellet was washed with 70% EtOH, centrifuged at 13000 g for 3 min at room temperature, air-dried, re-suspended in 200 µl of H_2_O with 20 µg/ml RNaseA and incubated at 50°C for 30 min.

### 2.3 Split ubiquitin screen

Total RNA was extracted from *Arabidopsis thaliana* Col-0 seedlings 15 days after sowing and from senescing rosette leaves 43 days after sowing as described (Downing et al., 1992), followed by purification of polyadenylated mRNA using oligo (dT)-cellulose Type 7 (Amersham Biosciences). cDNA synthesis, entry cDNA library construction, and cloning into pDONR222 were performed with the CloneMiner™ Library Construction Kit (ThermoFisher Scientific). The prey library for expression of NubG-cDNA fusion proteins in yeast was generated in pNubG-X (TAIR stock CD3-1737), a modified pNXgate32-3HA vector (Obrdlik et al., 2004). The entry cDNA library was transferred into the attR-sites of pNubG-X using the LR Clonase™ enzyme mix (ThermoFisher Scientific). Entry cDNA library and prey library plasmid DNA was isolated using the QIAfilter Plasmid Mega Kit (Qiagen).

The Cub-NRT1.5 bait vector was constructed by amplifying the *NRT1.5* (At1g32450) full-length coding sequence (CDS) including the stop codon on plasmid pcAcNRT1.5-9 (Drechsler et al., 2015) with Phusion High-Fidelity DNA Polymerase (ThermoFisher Scientific) and inserting it into PstI-SacII-digested pBT3-N plasmid (Dualsystems Biotech, Zürich, Switzerland). For the NRT1.5-Cub bait vector, the *NRT1.5* CDS lacking the stop codon was amplified and cloned into HindIII-digested pBT3-C plasmid (Dualsystems Biotech, Zürich, Switzerland). Each bait vector was transformed by the LiOAc/ssDNA/PEG method (Gietz and Schiestl, 2007) into yeast strain THY.AP4 [*MATa ura3 leu2 lexA::lacZ::trp1 lexA::HIS3 lexA::ADE2*], followed by transformation with the prey library plasmids. The split-ubiquitin screen was done following the DUALmembrane Kit 3 protocol (Dualsystems Biotech, Zürich, Switzerland). Colonies appearing after 5 days of growth on selective medium yeast nitrogen base (YNB-trp-leu-his-ade) supplemented with 5 mM 3-AT (3-amino-1,2,4-triazole) were propagated. For confirmation, the prey plasmids were recovered from the yeast, transformed in *E. coli* DH10B, reisolated, and retransformed into bait vector-carrying THY.AP4 cells. Confirmed prey plasmids expressing NRT1.5-interacting proteins were sequenced to identify the corresponding gene.

### 2.4 Split ubiquitin assay

pDONR222-NRT1.5 was generated by amplifying the full-length *NRT1.5* CDS lacking the stop codon from *A. thaliana* Col-0 cDNA using attB1/attB2 Gateway extension primers and Phusion High-Fidelity DNA Polymerase and inserting it into pDONR222. pDONR222-NRT1.5^G209E^ was generated from pDONR222-NRT1.5 by replacing the G^209^ codon GGA with GAA (➔E^209^) using the Q5-site-directed mutagenesis kit (New England Biolabs). pDONR222-AHA2 was constructed by recombining a synthetic attB1-AHA2-attB2 fragment (ThermoFisher/Invitrogen GeneArt Strings DNA Fragments) into pDONR222. The yeast expression vectors for NRT1.5-Cub and NRT1.5^G209E^-Cub were generated by LR reactions between pBT3-C and pDONR222-NRT1.5 and pDONR222-NRT1.5^G209E^, respectively. The expression vector for Nub-AHA2 was constructed by mobilizing the *AHA2* CDS from pDONR222-AHA2 into pNubG-X. Yeast strain THY.AP4 [*MATa ura3 leu2 lexA*::*lacZ*::*trp1 lexA*::*HIS3 lexA*::*ADE2*] was co-transformed with bait and prey vectors and positive colonies were selected after 2 days of growth at 30°C on YNB-trp-leu medium. Protein-protein interaction was detected by 4 days of growth on selective YNB-trp-leu-his-ade medium supplemented with 20 mM 3-AT.

### 2.5 Bi-molecular fluorescence complementation assay

The full-length CDS of *NRT1.5* with flanking attB1/attB4 Gateway attachment sites and of *AHA2* with flanking attB3/attB2 sites were amplified from *A. thaliana* Col-0 cDNA and recombined into pDONR221 P1-P4 and pDONR221 P3-P2, respectively. pDONR221p1-p4-NRT1.5^G209E^ was generated by site-directed mutagenesis of pDONR221p1-p4-NRT1.5. The three CDS cassettes were transferred into destination vector pBiFC-2in1-NN (Grefen and Blatt, 2012) by LR reaction. pBiFC-2in1-NN was a gift from Christopher Grefen (Addgene plasmid # 105111; http://n2t.net/addgene:105111). The vector pBiFC-2in1-NN includes a gene for expression of the soluble monomeric red fluorescent protein (mRFP1) as a reference marker for transformation and protein expression where the mRFP fluorescence signal visualizes the cytoplasm and the lumen of the nucleus. A pBiFC-2in1-NN construct with unfused nYFP and NRT1.5 fused cYFP was generated as a negative control. *Agrobacterium tumefaciens* GV3101::pMP90 cells (Koncz and Schell, 1986) were transformed by electroporation at 2.2 kV for 5 ms with the respective plasmid constructs and incubated with rifampicin (50 mg/ml), gentamycin (25 mg/ml), spectinomycin (75 mg/ml), and kanamycin (50 mg/ml) at 28°C for 2 days. Cultures were resuspended at OD_600 nm_ = 0.05 in infiltration solution (10 mM MES, pH 5.6, 10 mM MgCl_2,_ and 150 µM acetosyringone), incubated for 2 h at room temperature, and infiltrated into *Nicotiana benthamiana* abaxial epidermis cells (8-week old plants) with a syringe. Protein-protein interactions were detected two days post infiltration (dpi) by confocal microscopy.

### 2.6 Yeast growth assays

The full-length CDS of *AtNRT1.5* and *AtAHA2* were amplified with primers containing BamHI/HindIII restriction sites. *AHA2* was cloned in the yeast expression vector p425-TEF, and *NRT1.5* was cloned in p426-TEF (Mumberg et al., 1995). p426-TEF-NRT1.5^G209E^ was generated by site-directed mutagenesis of p426-TEF-NRT1.5. The expression plasmids were transformed into the control yeast strain BY4741 [*MAT*a *his3Δ leu2Δ met15Δ ura3Δ*] (Brachmann et al., 1998) and its derivative BYT12 [*trk1Δ trk2Δ*] which lacks the two major K^+^ uptake transporters (Petrezselyova et al., 2010). Transformants were precultured overnight in SD-leu-ura medium supplemented with 0.1 M KCl. The next day, the cells were washed with deionized water and resuspended to an OD_600 nm_ of 1, and spotted on SD-leu-ura plates. The hygromycin B (HygB) sensitivity test was performed according to Petrezselyova et al. (2010) and Zimmermannova et al. (2021). 20 µl of cell suspension (OD_600 nm_ = 1) were spotted on HygB gradient plates (0 to 0.5 g/l HygB, SD-leu-ura, 0.1 M KCl) and incubated at 30°C for two days.

### 2.7 Complementation of *nrt1.5* plants with NRT1.5 and NRT1.5^G209E^

The eGFP coding sequence lacking the initial ATG codon was amplified with overhangs to be cloned by Gibson assembly into pTkan+-PHO1NRT1.5 vector (Drechsler et al., 2015). The stop codon TAA of *NRT1.5* was removed by PCR. The pTkan+-PHO1NRT1.5 vector was opened by restriction enzyme digestion with PstI and ligated with the eGFP fragment using the NEBuilder HiFi DNA assembly kit (New England Biolabs). The pTkan+-PHO1NRT1.5^G209E^eGFP vector was constructed from pTkan+-PHO1NRT1.5eGFP using the Q5 Site-Directed Mutagenesis Kit (New England Biolabs).

The constructs were transformed in *nrt1.5* plants by the floral dip method (Clough and Bent, 1998). Two independently transformed *PHO1p::NRT1.5eGFP* and *PHO1p::NRT1.5^G209E^eGFP* lines in *nrt1.5* background that were homozygous for a single insertion of the transgene were used for this study.

### 2.8 Transient expression of NRT1.5eGFP and NRT1.5^G209E^eGFP in *Nicotiana benthamiana*

The coding sequences of eGFP, NRT1.5eGFP and NRT1.5^G209E^eGFP were amplified by PCR with the restriction enzyme attachment sites of BamHI and HindIII using pTkan+-PHO1NRT1.5eGFP and pTkan+-PHO1NRT1.5^G209E^eGFP as templates. The amplified products were ligated into Agrobacterium binary vector pCAMBIA containing the minimal CaMV 35S promoter sequence (Hey and Grimm, 2018). *Agrobacterium tumefaciens and Nicotiana benthamiana* transformations were performed as described for the BiFC assay.

### 2.9 RNA isolation, cDNA synthesis and qPCR

Total RNA from frozen *Arabidopsis thaliana* seedlings was extracted using the citric acid extraction method (Oñate-Sánchez and Vicente-Carbajosa, 2008) and DNase I treated according to the manufacturer’s instructions (New England Biolabs). First-strand cDNA was synthesized from 2 µg total RNA using RevertAid H Minus Reverse Transcriptase (ThermoFisher Scientific). Transcription analyses were performed with tissue from separately grown plants in three independent replicates. qPCR reactions were conducted on a CFX Connect real-time PCR detection system (Bio-Rad Laboratories) using the SYBR Green PCR Master Mix (ThermoFisher Scientific) and the thermal profile: 10 min 95°C and 40 times (15 sec 95°C, 1 min 60°C). For the subsequent melting curve analysis, the temperature was increased from 65 °C to 95 °C within 30 min. Expression values for each gene were normalized to the reference gene *UBQ10* (At4g05320), and relative expression levels were determined according to (Czechowski et al., 2005).

### 2.10 Extracellular acidification assay

Extracellular acidification was assayed as described by Haruta et al. (2010) with modifications. Seedlings were pre-germinated in 0.5 × MS medium for five days and then transferred to unbuffered 0.25 × MS-base containing either HK (10 mM K^+^, 10 mM NO_3_^-^) or 0K (0 mM K^+^, 1 mM NO_3_^-^) fertilizer for another five days. The ten-day-old seedlings were incubated in 200 µl modified 0.25 × MS media lacking MES (pH 5.5, unbuffered) with 30 µg/ml fluorescein isothiocyanate-dextran (FITC-dextran) (M_r_ 10,000, Sigma-Aldrich) for 16 h in long-day conditions (16 h/8 h light/dark, 22°C, light intensity 120 µmol m^-2^ s^-1^, relative humidity 40%). Excitation was at 485 nm and fluorescence was detected at 535 nm in a Power-Wave HT microplate spectrophotometer (BioTek). For pH determination, a calibration curve was generated with commercial MS media adjusted to pH values ranging from 4.5 to 7.5.

### 2.11 Live-cell imaging using confocal laser scanning microscopy

Confocal laser scanning microscopy (CLSM) of abaxial tobacco leaf epidermis cells was performed with a Leica TCS SP5 AOBS Confocal Microscope equipped with 20 x and 63 x water immersion objectives (Leica Microsystems). Simultaneous excitation of YFP (λ_ex_ = 488 nm, λ_em_ = 520-553 nm), mRFP (λ_ex_ = 561 nm, λ_em_ = 608-642 nm) and chlorophyll (λ_ex_ = 561 nm, λ_em_ = 642-702 nm) was achieved. The 514 nm argon laser was used to detect YFP and the 561 nm diode-pumped solid-state laser for mRFP and chlorophyll. Post-acquisition image processing and fluorescence quantification was performed using the Leica LAS AF software.

## 3 Results

### 3.1 The K^+^/NO_3_^-^ transporter NPF7.3/NRT1.5 interacts with the proton pump AHA2

To investigate whether NRT1.5 interacts with other PM proteins, we carried out a split-ubiquitin membrane yeast two-hybrid screen (Stagljar et al., 1998; Thaminy et al., 2003). As *AtNRT1.5* is highly expressed in seedling roots and senescing leaves (van der Graaff et al., 2006; Lin et al., 2008; Zheng et al., 2016), we used as prey two *A. thaliana* cDNA libraries from these tissues and screened them with two bait constructs carrying the Cub-LexA-VP16 reporter module at the N- or C-terminus of NRT1.5, respectively. From the primary NRT1.5-interactor candidates we selected proteins that are known to be localized in the PM, to be expressed in pericycle cells of Arabidopsis roots, and to be associated with ion- or protein-transport. To confirm the interaction with NRT1.5, the full-length coding sequences of the candidates were amplified from *A. thaliana* Col-0 cDNA, fused to NubG, and used as prey for the NRT1.5 bait in targeted split-ubiquitin assays. Five putative NRT1.5-interactors were confirmed in this assay (**Supplementary Table 3**).

**Figure 1:**
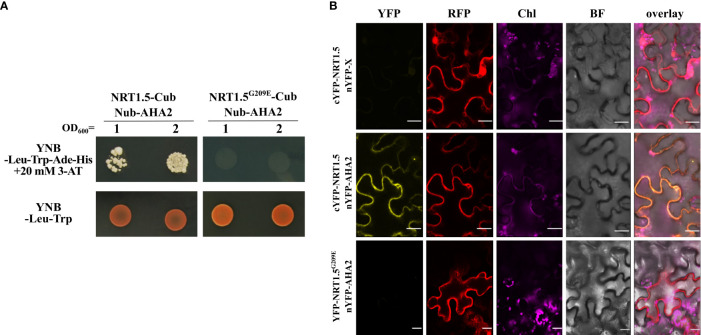
Protein-protein interaction of NRT1.5 and AHA2. **(A)** THY.AP4 cells were transformed with NRT1.5 or NRT1.5^G209E^ fused to Cub and AHA2 fused to Nub. To detect protein-protein-interaction, 12 µl yeast cell culture with an OD_600 nm_ =1 and OD_600 nm_ =2, respectively, were dropped on YNB-Leu-Trp-His-Ade medium supplemented with 20 mM 3-amino-1,2,4-triazole (3-AT) and on YNB-Leu-Trp medium as growth control. The addition of 3-AT reduces non-specific yeast growth not based on protein-protein interactions. Plates were incubated at 30°C for three days and then photographed. **(B)** Confirmation of the interaction of NRT1.5 with AHA2 by bimolecular complementation assay. Shown are confocal microscopy images of *N. benthamiana* epidermis cells two days post-infiltration co-expressing non-fused nYFP, cYFP-NRT1.5 and RFP (top row), nYFP-AHA2, cYFP-NRT1.5 and RFP (center row), and nYFP-AHA2, cYFP-NRT1.5^G209E^ and RFP (bottom row). Column YFP: YFP fluorescence in yellow indicates interaction between proteins. Column RFP, RFP fluorescence in red visualizes the cytoplasm and the lumen of the nucleus as expression control. Column Chl, autofluorescence of chlorophyll (represented in violet). Column BF, bright field image. Column overlay: overlay of all 4 channels. Scale bar = 20 µm.

Among them was the PM proton ATPase 2 (AHA2) whose interaction with NRT1.5 we investigated in more detail in this study. Co-expression of NRT1.5-Cub and NubG-AHA2 in the auxotrophic yeast strain THY.AP4 led to yeast growth in a medium lacking adenine and histidine, indicating that NRT1.5 directly interacts with AHA2 (**Figure 1A**).

To evaluate the protein-protein interaction in plant cells, a bi-molecular fluorescence complementation assay was carried out in *Nicotiana benthamiana* cells. Co-expression of the cYFP-NRT1.5 and AHA2-nYFP fusion proteins reconstituted YFP fluorescence, suggesting the interaction of NRT1.5 and AHA2 at the plant PM (**Figure 1B**).

### 3.2 The Gly^209^ of NTR1.5 is critical for AHA2 interaction and affects NRT1.5 function but not its cellular localization in plants

Li and colleagues (2017) had discovered that the low-K^+^-sensitive *A. thaliana* mutant *lks2* carries a Gly^209^ to Glu (G209E) substitution in NRT1.5 and that the NRT1.5^G209E^ mutant protein is compromised in K^+^ efflux in *Xenopus laevis* oocytes and in yeast cells. We observed that the mutant protein does neither interact with AHA2 in the yeast assay (**Figure 1A**) or in tobacco cells (**Figure 1B**). This corroborates the specificity of the NRT1.5-AHA2 interaction and demonstrates that Gly^209^ of NRT1.5 is critical for the interaction with AHA2.

Previously it was shown that, as a consequence of an impaired K^+^ root-to-shoot transport, the *nrt1.5* mutant displays impaired root architecture and develops an early senescence phenotype when plants grow in media lacking potassium and nitrate (Drechsler et al., 2015; Zheng et al., 2016). Additionally, *nrt1.5* seedlings exhibit a reduced Hygromycin B (HygB) sensitivity due to a reduction in the PM potential (Drechsler et al., 2015).

The NRT1.5 Gly^209^ residue is highly conserved in NRT1.5 homologs in other plant species as well as in the 16 NRT1 family members in *A. thaliana* (**Supplementary Figure 3**). To evaluate if *NRT1.5^G209E^* expression in root cells affects NRT1.5 function, we carried out a complementation assay of the *nrt1.5* mutant with NRT1.5^G209E^. *nrt1.5* mutant plants were transformed with a vector containing the promoter of the PHOSPHATE1 (*PHO1*) gene, and the *NRT1.5* or *NRT1.5^G209E^* coding region lacking the stop codon fused to a C-terminal eGFP fluorescent tag.

The 1.6 kb *PHO1* promoter (*PHO1p*) served as substitute for the *NRT1.5* promoter in *nrt1.5* complementation experiments, because expressing the *NRT1.5* coding region under the control of the 2.4 kb upstream region of *NRT1.5* or the ubiquitous 35S CaMV promoter does not achieve complementation of the *nrt1.5* mutant (Drechsler et al., 2015). *PHO1* (*At3g23430*) was identified as a gene whose expression is like that of *NRT1.5* primarily directed to the root vasculature in Arabidopsis plants (Hamburger et al., 2002). Transgenic plants with the *NRT1.5* coding region fused behind the *PHO1* promoter expressed almost wild-type levels of *NRT1.5* transcripts in the roots (Drechsler et al., 2015). Homozygous *PHO1p::NRT1.5eGFP* and *PHO1p::NRT1.5eGFP^G209E^* plants were evaluated phenotypically under K^+^/NO_3_^-^ deficiency and exposure to HygB.

**Figure 2:**
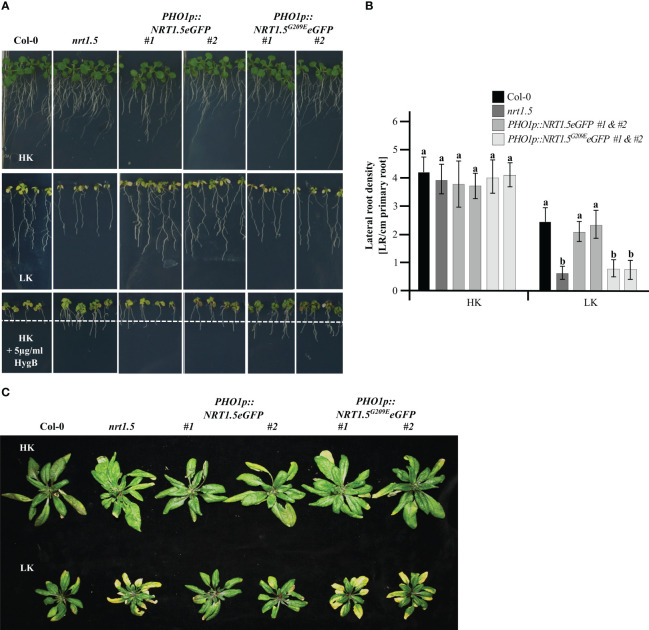
Phenotypes of the complementation lines *nrt1.5 PHO1p::NRT1.5eGFP* and *nrt1.5 PHO1p::NRT1.5^G209E^eGFP*. **(A)** Root phenotypes. Col-0, *nrt1.5*, two independent *nrt1.5, PHO1p::NRT1.5eGFP* and two independent *nrt1.5, PHO1p::NRT1.5^G209E^eGFP* plants were pre-germinated in 0.5 × MS medium for five days and transferred to plates with low nutrient medium (LK: 1 mM K^+^, 1 mM NO_3_^-^), high nutrient medium (HK: 10 mM K^+^, 10 mM NO_3_^-^) or HK + 5 µg/ml HygB medium for ten more days. The dotted white line represents the maximum root growth of Col-0 in HK + 5 µg/ml HygB. **(B)** Lateral root density quantification of plants in HK or LK conditions as in panel **(A)** Different letters indicate statistically significant differences (Tukey’s test) between mutants and Col-0 in the different treatments with *P <*0.05, (means ± SD, n =20). **(C)** Shoot phenotypes. Plants were pre-germinated on fertilized soil for seven days, transferred to unfertilized type soil, and supplemented with a one-half strength MS-based fertilization solution containing HK or LK (**Supplementary Table 2**) for 40 days.

The root growth assay indicated that *PHO1p::NRT1.5eGFP* plants can rescue the reduction in root density (**Figures 2A, B**) and the early senescence phenotype of *nrt1.5* plants under K^+^ and NO_3_^-^ deficiency (**Figure 2C**), corroborating previous studies of this complementation line without the fluorescent tag (Drechsler et al., 2015). In contrast, the transgenic *nrt1.5 PHO1p::NRT1.5^G209E^eGFP* plants retained the *nrt1.5* root and shoot phenotypes, demonstrating that the mutation G209E prevents complementation of the *nrt1.5* mutant. Expression of the NRT1.5 and NRT1.5^G209E^ proteins was confirmed by the detection of GFP signals in the transgenic plant roots and at the PM of tobacco epidermal cells (**Supplementary Figure 1**).

Hygromycin B (HygB) is used as an indicator of the PM potential status in plant and yeast cells (Haruta et al., 2010; Barreto et al., 2011; Haruta and Sussman, 2012). When the PM potential is reduced, the toxic drug is not able to enter the cells and the plants develop an enhanced HygB tolerance in comparison to wild-type, as was observed in *nrt1.5* mutant plants (Drechsler et al., 2015). The two independent *PHO1p::NRT1.5eGFP* lines were sensitive to 5 µg/ml HygB (reduction of root growth and yellow cotyledons), whereas the *PHO1p::NRT1.5^G209E^eGFP* lines were more resistant, indicating that the PM potential was restored upon expression of NRT1.5 in the *nrt1.5* mutant background (**Figure 2A**). In contrast, the NRT1.5^G209E^ mutant protein is not able to complement the *nrt1.5* mutant, most likely because of a disturbed ion transport function of mutant protein, consistent with heterologous expression results in *Xenopus laevis* oocytes (Li et al., 2017).

### 3.3 The proton pump AHA2 contributes to K^+^ translocation under limiting K^+^ and NO_3_^-^ supply

**Figure 3:**
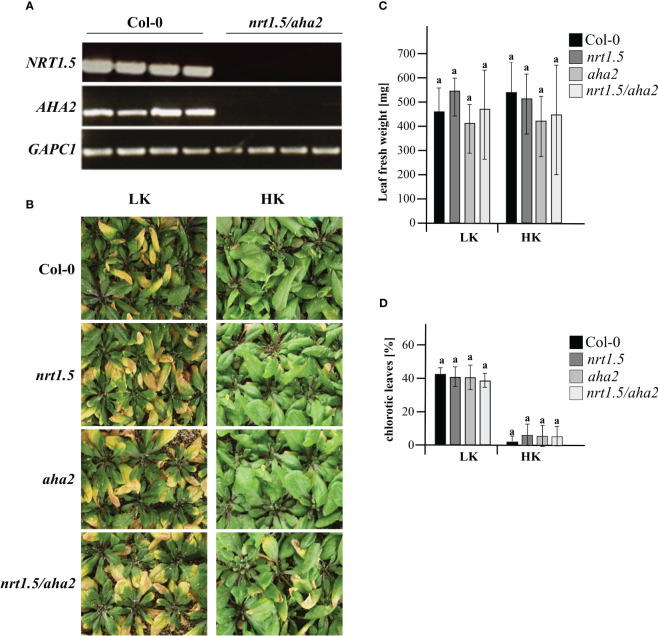
Phenotyping of wild-type and mutant plants under low (LK) and high nutrient (HK) conditions. **(A)** RT-PCR analysis of *NTR1.5* and *AHA2* transcripts in Col-0 and the *nrt1.5/aha2* double mutant. *GAPC1* (At3g04120) was used as reference housekeeping gene **(B)** Rosette phenotypes, **(C)** Leaf fresh weight, and **(D)** Fraction of chlorotic leaves (%) of Col-0, *nrt1.5*, *aha2*, and *nrt1.5/aha2* plants that were pre-germinated in fertilized soil for seven days and transferred to unfertilized soil type for another 30 days while watered with HK or LK medium, respectively. Shown are the means ± SD of the fraction of chlorotic leaves in 20 individual plants. Different letters above columns indicate statistically significant differences (Tukey’s test; P <0.05).

To investigate if the NRT1.5 interaction with AHA2 is required for nutrient root-to-shoot translocation in Arabidopsis, the *nrt1.5*/*aha2* double mutant was generated. No residual *NRT1.5* or *AHA2* transcripts were detectable by semi-quantitative RT-PCR in the double mutant (**Figure 3A**). When grown on unfertilized soil and watered with low-nutrient fertilizer (LK: 1 mM K^+^, 1 mM NO_3_^-^), wild-type, single, and double mutant plants showed a similar degree of early shoot chlorosis compared to plants grown under ample nutrient supply (HK: 10 mM K^+^, 10 mM NO_3_^-^) (**Figure 3B**), and no reduction in fresh weight (**Figure 3C**). Under ample nutrient supply, all plant lines developed chlorosis in less than 10% of leaves, whereas with low nutrient fertilization approximately 40% of the leaves showed leaves chlorosis (**Figure 3D**).

**Figure 4:**
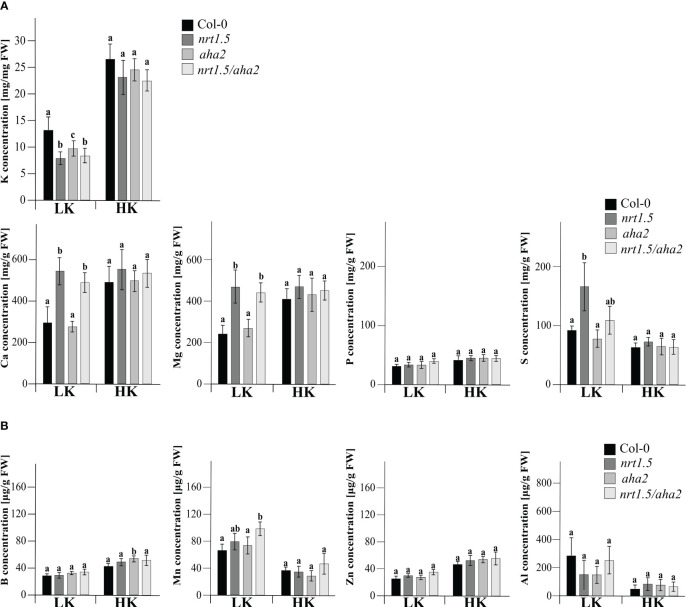
Elemental analysis by ICP-OES of Arabidopsis Col-0, *nrt1.5*, *aha2*, and *nrt1.5/aha2* plants grown under low (LK) and high (HK) nutrient conditions. **(A)** Macronutrient and **(B)** micronutrient concentration in shoots of 30 days-old Col-0, *nrt1.5*, *aha2*, and *nrt1.5/aha2* plants grown in LK and HK medium. Different letters above columns indicate statistically significant differences (Tukey’s test; P <0.05) between mutants and Col-0 (means ± SD, n =8).

In an earlier study it was shown that nitrate as well as total N concentrations in rosettes of *nrt1.5* mutant plants are similar as in Col-0 wild-type plants under both low and high N fertilization regimes, whereas the K concentration in the mutants remained below 30% and 50% of the wild-type levels (Drechsler et al., 2015). We therefore determined here the concentration of mineral nutrients in 30 days old leaves of Col-0, *nrt1.5*, *aha2*, and *nrt1.5/aha2* plants by inductively coupled plasma optical emission spectrometry (ICP-OES). When fertilized with 10 mM potassium and nitrate (HK), the concentrations of none of the elements K, Ca, Mg, B, Mn, Zn, Al, P, and S differed between wild-type, *nrt1.5*, *aha2*, or *nrt1.5/aha2* leaves (**Figure 4**). In contrast, under limiting nutrient supply (LK) Ca, Mg, and S concentrations were increased in *nrt1.5* and *nrt1.5/aha2* leaves, indicating that the lack of NRT1.5 disturbs the distribution of these elements between roots and shoots, whereas K levels were significantly decreased in *nrt1.5*, *nrt1.5/aha2* and also *aha2* mutant plants. These results suggest that in low nutrient conditions both NRT1.5 and AHA2 are required for efficient K^+^ translocation to shoots, whereas the distribution of Ca, Mg, and S is not dependent on AHA2.

**Figure 5:**
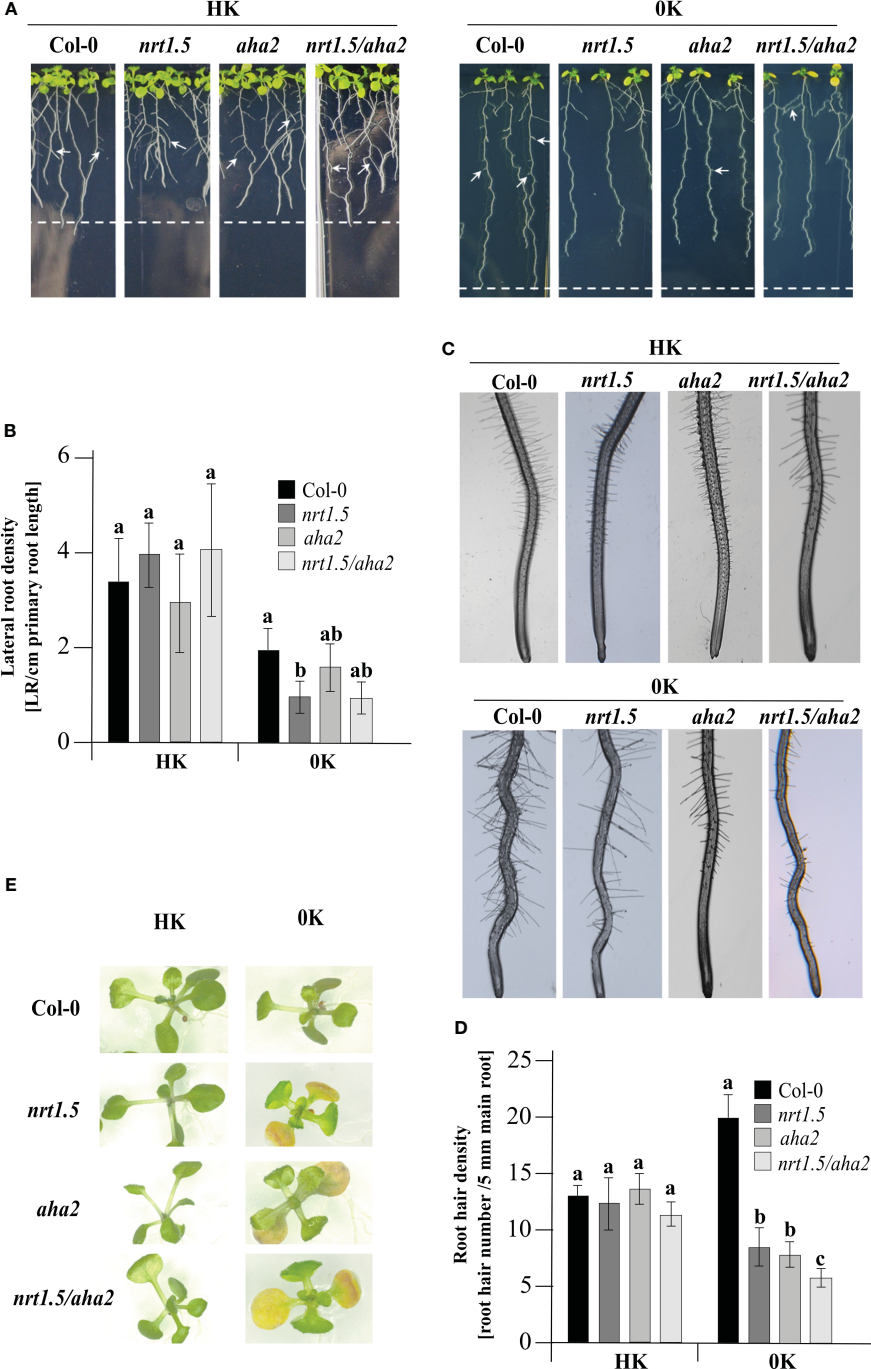
Phenotypes of Col-0, *nrt1.5*, *aha2*, and *nrt1.5/aha2* seedlings at 0K or HK supply. **(A)** Root phenotypes. Seedlings were pre-germinated in 0.5 × MS medium for five days and then transferred to 0K or HK media for fourteen days further. White arrows show lateral root formation. Dotted lines represent the maximum primary root growth of Col-0 in each condition. **(B)** Quantification of the density of lateral roots from **(A)** The number of lateral roots formed/main length of the primary root (cm) was plotted as lateral root density. Different letters indicate statistically significant differences (Tukey’s test) between mutants and Col-0 with *P <*0.05, (means ± SD, n ≥15). **(C)** Root hair phenotypes of seedlings in **(A)**. **(D)** Root hair density was determined as the number of hair roots in a 15 mm section from the starting point of the root tip under a light stereomicroscope (SZX12, Olympus) and quantified using Image J software. Results were expressed as the mean ± standard error. Different letters indicate statistically significant differences (Tukey’s test) between mutants and Col-0 with *P <*0.05, (means ± SD, n =15). **(E)** Shoot phenotypes of seedlings in A after a week of growth in the conditions indicated.

The contribution of NRT1.5 to K^+^ translocation from roots to shoots under low K^+^ supply was reported by Li and colleagues before (Li et al., 2017). In addition, under low K^+^ in combination with low nitrate conditions, the *nrt1.5* mutant exhibits leaf chlorosis and a reduced lateral root density (Zheng et al., 2016; Li et al., 2017). To investigate whether AHA2 is also involved in nutrient translocation, a seedling growth test was carried out. In the presence of HK root development did not differ in Col-0 wild-type and the three mutant plants, where all plants showed the same lateral root (**Figures 5A, B**) and root hair density (**Figures 5C, D**). In 0K medium, *nrt1.5* mutant seedlings show a significant reduction in lateral root density. In the *nrt1.5/aha2* double mutant, the lateral root density is similarly reduced by trend, whereas in *aha2* seedlings it is similar to Col-0 (**Figure 5B**). However, in all three mutant lines and most prominently in the *nrt1.5/aha2* double mutant plants the density of root hairs is significantly reduced in 0K medium compared to wild-type (**Figure 5D**) and the cotyledons develop an early chlorosis phenotype (**Figure 5E**). These observations suggest that in addition to NRT1.5 also AHA2 is involved in nutrient translocation to shoots in young seedlings.

### 3.4 NRT1.5 and AHA2 expression influence the plasma membrane potential and pH homeostasis under low nutrient supply

NRT1.5 exports K^+^ from root parenchyma cells into the xylem vessels at weakly acidic extracellular pH conditions like in the xylem sap (Li et al., 2017). AHA2 is the major PM proton efflux pump in root cells (Harper et al., 1990; Haruta et al., 2010). Both transporters effectuate ion movement across the PM of root cells and thus affect the PM potential. In plants, PM H^+^-ATPases are responsible for the establishment of the cellular membrane potential (Sondergaard et al., 2004). To investigate if NRT1.5 and AHA2 cooperatively contribute to the maintenance of the PM potential, we indirectly measured this parameter in yeast and plant cells overexpressing or lacking NRT1.5 and AHA2.

**Figure 6:**
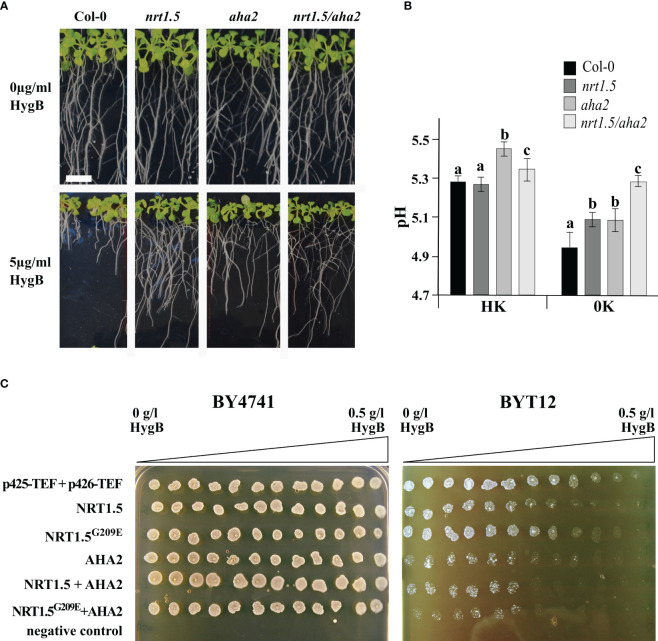
pH and plasma membrane potential are influenced by NRT1.5 and AHA2 expression. **(A)** Root growth phenotypes of Col-0, *nrt1.5*, *aha2*, and *nrt1.5/aha2* in response to 5 µg/ml HygB. 5-day-old seedlings were transferred to 0.5 × MS medium pH 5.5 supplemented with or without 5 µg/ml HygB for fourteen more days. The white bar represents 1 cm. **(B)** Extracellular pH measurements in Col-0, *nrt1.5*, *aha2, nrt1.5/aha2* roots under HK and 0K nutrition supply. Ten days-old seedlings were incubated with 0.25 x MS liquid medium supplemented with 30 µg/ml of FITC-dextran for 16 h. Media pH was determined by a fluorescence standard curve with a pH range from 4.5 to 7.5. Different letters indicate statistically significant differences (Tukey’s test) between mutants and Col-0 with *P*<0.05, (means ± SD, n =12). **(C)** BY4741 wild-type and BYT12 mutant yeast strains were transformed with empty expression vectors (p425-TEF and p426-TEF) or expression constructs for the indicated proteins. Twelve 20 µl drops of untransformed (negative control) or transformed yeast cell suspension (OD_600 nm_=1) were spotted along the HygB gradient (from 0 to 0.5 g/l) on SD-leu-ura pH 6.0, 0.1 M KCl plates and incubated at 30°C for two days. p425-TEF and p426-TEF are the empty expression vectors.

In yeast cells and plant roots, the uptake of the toxic cationic drug Hygromycin B (HygB) depends on the PM potential. Sensitivity to HygB is often linked to changes in the PM potential, which can be provoked by alterations in K^+^ homeostasis in *S. cerevisiae* (Barreto et al., 2011) or alterations in H^+^ homeostasis in *A. thaliana* (Haruta and Sussman, 2012). We observed that the growth of Arabidopsis Col-0 seedling roots was highly sensitive to 5µg/ml HygB. In contrast, root growth of the *nrt1.5*, *aha2*, and *nrt1.5*/*aha2* mutants is much less sensitive to HygB (**Figure 6A**), implying that the lack of *NRT1.5* and *AHA2* leads to membrane depolarization.

In a HygB sensitivity assay with the yeast mutant BYT12, which lacks the two main K^+^-uptake transporters Trk1 and Trk2, the expression of *NRT1.5* causes hyperpolarization of the yeast PM, indicating that NRT1.5 is active in yeast cells (Drechsler et al., 2015). To investigate whether NRT1.5 and AHA2 affect the PM potential, both genes were individually or co-expressed in wild-type BY4741 and mutant BYT12 cells. Subsequently, yeast growth was monitored in plates containing high K^+^ concentration (0.1 M), which supports growth of BYT12 cells, and a HygB concentration gradient. Overexpression of the *NRT1.5^G209E^* mutant protein was included in the assay to evaluate the impact of the mutation in yeast. The expression of *NRT1.5* in BYT12 cells caused increased sensitivity to high HygB concentrations (**Figure 6C**), as previously reported by Drechsler et al. (2015). Expression of the mutant *NRT1.5^G209E^* led to partial growth recovery of BYT12 cells, suggesting the mutation G209E affects the NRT1.5 ion translocation function in yeast. The expression of *AHA2* in BYT12 cells results in increased HygB sensitivity compared to BYT12 cells expressing *NRT1.5*, suggesting that the PM potential is strongly influenced by the expression of *AHA2.* BYT12 cells expressing NRT1.5 and AHA2 exhibited the same sensitivity to HygB as cells expressing AHA2 alone (**Figure 6C**). Thus, this assay does not indicate a biological interaction between NRT1.5 and AHA2 in yeast cells.

Plant PM H^+^-ATPases use energy from ATP hydrolysis to pump protons from the cytoplasm to the extracellular space, thus creating and maintaining a negative membrane potential at the cytosolic side and a transmembrane pH gradient that acidifies the extracellular space (reviewed by Sondergaard et al., 2004; Falhof et al., 2016) and controls the transport of nutrients across the PM (Cosse and Seidel, 2021). To investigate if NRT1.5 and AHA2 cooperatively influence the extracellular pH, we incubated Col-0, *nrt1.5*, *aha2*, and *nrt1.5/aha2* seedlings in media with different K^+^ and NO_3_^-^ concentrations and determined the pH using the fluorescent dye FITC-dextran. The assay detects pH differences at the Arabidopsis root surface with a sensitivity that is comparable to that of pH-sensitive microelectrodes (Hoffmann and Kosegarten, 1995; Pitann et al., 2009; Haruta et al., 2010; Haruta et al., 2018). At high nutrient supply (HK), the extracellular pH of Col-0 and the *nrt1.5* mutant roots were similar (pH ≈5.3), whereas *aha2* roots led to an increase to almost pH 5.5 (**Figure 6B**). An increase in extracellular pH indicates an impaired PM proton-secretion activity and has previously also been observed in two other *aha2* mutants (Haruta et al., 2010). The *nrt1.5/aha2* double mutants showed an intermediate increase to pH ≈5.4. Wild-type roots showed at 0K supply a reduction of the extracellular pH to ~4.9, supporting that low nutrient availability induces acidification of the extracellular space or rhizosphere (Houmani et al., 2015), presumably due to increased activity of PM H^+^-ATPases. In contrast, extracellular acidification by *nrt1.5*, *aha2*, and *nrt1.5/aha2* mutants was reduced under these conditions, where the double mutant showed the highest reduction of acidification to pH ≈5.3. These results are consistent with the hypothesis that in wild-type plants under altered K^+^ and NO_3_^-^ supply NRT1.5 and AHA2 interact and mutually activate each other. As AHA2 exports more protons than NRT1.5 imports, the acidification of the extracellular space is relatively strong. In the mutants the mutual induction of both proteins is absent, resulting in a slightly higher extracellular pH (**Figure 6B**).

The only subtle phenotypic differences of the *aha2* and *nrt1.5/aha2* double mutants relative to the *nrt1.5* mutant prompted us to investigate whether other H^+^-ATPase family members are upregulated when AHA2 is lacking, we examined the gene expression of all H^+^-ATPase genes (*AHA1*-*AHA11*) in Col-0, *nrt1.5*, *aha2*, and *nrt1.5/aha2* seedlings relative to AHA2 and their response to low nutrient supply. Except for *AHA1*, which exhibits a similar absolute expression level as *AHA2*, all other gene family members are expressed at 10- to 10,000-fold lower levels than *AHA2* (**Supplementary Figure 2A**). These results are confirmed by transcriptome data sets in the Arabidopsis eFP Browser (https://bar.utoronto.ca/efp_arabidopsis/cgi-bin/efpWeb.cgi; Winter et al., 2007) which show that *AHA1* and *AHA2* are the only gene family members that are significantly expressed in seedlings or seedling roots. Under low nutrient supply *AHA2* is ~2fold and *AHA1* is ~4fold upregulated in the *nrt1.5* mutant. Surprisingly though, no upregulation of *AHA1* is observed in the *nrt1.5/aha2* double mutant plants. In the *aha2* mutant we observe some upregulation of *AHA6* and *AHA9* (**Supplementary Figure 2B**), however, due to the extremely low basic expression of these genes in seedlings it is very unlikely that they could compensate for the lack of AHA2 in the *aha2* mutant.

## 4 Discussion

### 4.1 NRT1.5 and AHA2 coordinately control the H^+^ and K^+^ homeostasis in plants

In this study, we demonstrate that the H^+^/K^+^-antiporter NRT1.5 interacts *in vivo* with the plasma membrane H^+^-ATPase AHA2 and that both proteins contribute to K^+^ transport under K^+^ and NO_3_^-^ deficiency. Our results support the assumption that proton-motive force generated by AHA2 energizes the K^+^ transport across the PM by NRT1.5.

AHA2 is expressed in many tissues, most strongly in different root cells, stomatal guard cells, and mesophyll cells (Alexandersson et al., 2004; Sondergaard et al., 2004; Młodzińska et al., 2015; Falhof et al., 2016). In roots it is the major proton pump and, like NRT1.5, it is found in the PM of pericycle cells, which is a prerequisite for a direct interaction between the two proteins (Nicolson, 2014). A close link between proton transport and K^+^ fluxes at the PM has already been concluded by the induction of the K^+^ transporters HAK5 and CHX17 under K^+^ starvation in wild-type (Cellier et al., 2004; Gierth et al., 2005) and *aha2* mutant seedlings (Haruta and Sussman, 2012). Moreover, K^+^ uptake under low K^+^ conditions is promoted by the direct interaction between AHA2 and the receptor-like protein kinase BAK1 (Wang et al., 2022). Since the electrical membrane potential and chemical pH gradient generated by the proton-pumping ATPase AHA2 are essential for the function of the majority of plant PM transporters, it is not surprising that AHA2 is regulated by and interacting with many physiological parameters and protein factors (reviewed by Falhof et al., 2016; Michalak et al., 2022). *AHA2* expression is affected by changing nitrate supply (Młodzińska et al., 2015) and phosphorus deficiency (Yuan et al., 2017). An additional layer of complexity of AHA2 regulation is added by post-translational control of the AHA2 protein activity. AHA2 can be phosphorylated at its C-terminal end by at least two kinases, which either enables or prevents binding of activating 14-3-3 proteins (Fuglsang et al., 1999; Fuglsang et al., 2007; Haruta et al., 2015). Recently, AHA2 was identified to be a central node in a protein-protein interaction network that undergoes highly dynamic protein-protein interaction assembly and disassembly processes upon nitrogen starvation (Gilbert et al., 2021).

We found that under low potassium and nitrate supply the lack of NRT1.5 and/or AHA2 in Arabidopsis seedlings triggered a decrease of K^+^ in leaves (**Figure 4A**), and a strong reduction in root hair density relative to Col-0 wild-type plants (**Figure 5**). Modulation of root architecture by nutrient availability is a well-known and diverse response in plants (Forde and Lorenzo, 2001; Gruber et al., 2013). It is likely that AHA2 indirectly contributes to these root morphology alterations as it facilitates an acidic cell wall environment by extrusion of protons to the apoplast, which is essential for cell wall expansion and cell growth (Pacifici et al., 2018; Sze and Chanroj, 2018).

Eliminating or overexpressing *NRT1.5* and *AHA2* in Arabidopsis and yeast, respectively, caused an alteration of the PM potential (**Figures 6A, C**). In AHA2-lacking seedlings grown under normal N and K^+^ supply the extracellular pH in the medium is slightly elevated (**Figure 6B**), as was also observed in other studies (Haruta et al., 2010). When wild-type plants face K^+^ deprivation, the extracellular pH drops sharply, indicating an increased H^+^ export or reduced H^+^ import or both. A reduced H^+^ import could results from down-regulation of K^+^/H^+^ antiporters like NRT1.5. In *aha2* and *nrt1.5* mutant seedlings the drop in extracellular pH is lower under K^+^ deprivation compared to wild type plants, and in the *nrt1.5/aha2* double mutant no more pH alteration is observed. This indicates that under these conditions NRT1.5 and AHA2 cooperatively modulate the extracellular pH, which is supposedly accomplished by the direct interaction of the two proteins.

In a screen for low-K^+^-sensitive Arabidopsis mutants Li et al. (2017) identified the *lks2* mutant, in which a single-nucleotide mutation causes the substitution of Gly^209^ to Glu (G209E) in NRT1.5. The NRT1.5^G209E^ protein is not able to complement the *nrt1.5* phenotype in roots or shoots of Arabidopsis (**Figure 2**), and its expression in yeast does not phenocopy the PM potential status of cells expressing NRT1.5 (**Figure 6**). As all Arabidopsis NRT1 proteins contain either Gly, Ala or Val at the NRT1.5^G209^ homologous position (**Supplementary Figure 3B**), which is also conserved in NRT1.5 homologs from other plant species (**Supplementary Figure 3A**), it seems likely that an uncharged amino acid at this position is functionally crucial for NRT1 family transporters. Gly^209^ is not exposed at a cytosolic or extracellular loop of the transporter, but is located in the center of transmembrane span 5. It therefore less likely that Gly^209^ is directly involved in the interaction with AHA2, but it rather is critical for the structural integrity of NRT1.5.

### 4.2 Different proton pumps from the H^+^-ATPases family may contribute to the cellular pH control under low K^+^ and NO_3_^-^ supply

In the *Arabidopsis thaliana* genome 11 PM H^+^-ATPase (AHA) encoding genes and one pseudogene (*AHA12*) were identified (reviewed in Gaxiola et al., 2007). AHA1 and AHA2 are the predominantly expressed isoforms in Arabidopsis seedlings, leaves and roots, but also low levels of AHA3, AHA4, and AHA11 were detected by transcriptomics and/or mass spectrometry (Harper et al., 1990; Alexandersson et al., 2004; Haruta et al., 2010). In the root endodermis AHA4 was shown to contribute to ion homeostasis and nutrient transport (Vitart et al., 2001). AHA6 and AHA9 are essential for pollen tube growth and fertility (Hoffmann et al., 2020). Some PM H^+^-ATPases have been reported to be transcriptionally induced under abiotic stresses including nutrition, light, and salinity (Gévaudant et al., 2007; Caesar et al., 2011; Yuan et al., 2017; Ando and Kinoshita, 2018). However, reports on specific physiological functions of Arabidopsis AHA family members are sparse, presumably because of functional redundancy of the gene family members (Gaxiola et al., 2007; Haruta et al., 2010). For example, AHA1 can functionally complement for AHA2 (Rodrigues et al., 2014).

In *aha2* and *nrt1.5/aha2* mutants we observed under K^+^ deprivation conditions up-regulation of *AHA4, AHA6* and *AHA9* transcription (**Supplementary Figure 2**). However, as the regulation of these genes under LK- versus HK-conditions was inconsistent in *aha2* and *nrt1.5/aha2* plants and even the induced expression level of these genes remained more than 100 fold lower than that of *AHA2*, we have no evidence for complementation of the lack of AHA2 in the mutants by other AHA family members.

Arabidopsis NRT1.5 is not only a nitrate transporter involved in long-distance NO_3_^-^ transport from root to shoot, but under NO_3_^-^ deficiency also a potassium transporter loading K^+^ into the xylem. Here, the identification of the proton pump AHA2 as an interactor partner of NRT1.5 reveals a novel regulatory level in the control of K^+^ and H^+^ homeostasis in Arabidopsis.

## Data availability statement

The original contributions presented in the study are included in the article/**Supplementary Material**. Further inquiries can be directed to the corresponding author.

## Author contributions

FS: Conceptualization, Investigation, Methodology, Writing – original draft. RK: Conceptualization, Funding acquisition, Project administration, Supervision, Writing – review & editing.
