# Supplementary material for: The K+ transporter NPF7.3/NRT1.5 and the proton pump AHA2 contribute to K+ transport in Arabidopsis thaliana under K+ and NO3 - deficiency
Source: Front Plant Sci. 2023 Nov 10;14:1287843. doi: 10.3389/fpls.2023.1287843 (PMC10690419; doi:10.3389/fpls.2023.1287843)
Supplement: Supplementary file 1 [file DataSheet_1.pdf]

Supplementary Material

The K^+ transporter NPF7.3/NRT1.5 and the proton pump AHA2 contribute to K^+ transport in *Arabidopsis thaliana* under K^+ and NO_3^- deficiency

Florencia Sena[1,2,3], Reinhard Kunze[1*]

[1]Institute of Biology/Applied Genetics, Dahlem Centre of Plant Sciences, Freie Universität Berlin, Berlin, Germany

[2]Laboratory of Apicomplexan Biology, Insitut Pasteur Montevideo, Montevideo, Uruguay

[3]Laboratorio de Bioquímica, Facultad de Agronomía, Universidad de la República, Montevideo, Uruguay

*** Correspondence:** reinhard.kunze@fu-berlin.de

Supplementary Table 1: Oligonucleotides used in this work. Restriction enzyme sites, Gateway cloning and Gibson cloning adaptor sequences in forward (F) and reverse (R) primer sequences are shown in lowercase letters. Oligonucleotide sequences for qPCR analyses were calculated by the QuantPrime online tool (Arvidsson et al., 2008).

Template	Sequence (5'- 3')
**qPCR**	
NRT1.5	F: TGCTGGCATCGTCATTCTTCTG<br>R: AGCACCAAGTTCACTCCAACTCC
AHA2	F: AGAAGAGCTGAGATCGCTAGGC<br>R: TGTCCAAGCCCTTTAGCTTCACG
UBQ10	F: GGCCTTGTATAATCCCTGATGAATAAG<br>R:AAAGAGATAACAGGAACGGAAACATA
AHA1	F: GCCAGCTTGTTTGACAACAGGAC<br>R: TTTGGCTGCAGACCGTGCAATG
AHA3	F: CCGTTACATACTAGCCGGAACAGC<br>R: TTGCTTAGTGGTAAACGCAGTCC
AHA4	F: GGAGAGCTGAAATTGCTAGGTTGC<br>R: ACCCAACTGAAATCAGCTTGCAC
AHA5	F: AAGCCAGGCTCTCATCTTCGTC<br>R: ACTGCTATAAATGTCGCCACCAG
AHA6	F: GAAACGTGCAGAAGTTGCTAGGC<br>R: TACCGACTCAACGTGGCCTTTG
AHA7	F: CAGCCAAGCGTTGATCTTCGTG<br>R: CCGATATCACCGATGCAACCAG
AHA8	F: TGGCGACTGAATTCTCGTGGGATG<br>R: TTCTTCCACAGGGATCCGCTCAAG
AHA9	F: TGTTGAACGTCCTGGCTTCTGG<br>R: ACCGCAATCAAAGTAGCAATCAGC
AHA10	F: TGCCTTCATTCTTGCTCAACTTGC<br>R: AGCAAAGCTGATGTTGGCATACAC
AHA11	F: CATTGGCGGTATCCCAATTGCC<br>R: CCTCTTGGTTATTGCTCCCTGCTG
**Genotyping PCR**	

LB_Gabi	CCATTTGGACGTGAATGTAGACAC
LP_NRT1.5	CTCGAAGATTGCGTTTTTCAG
RP_NRT1.5	CCCGATGAGTGAGTATTGTGG
LP_AHA2	TGACAAAACCGGGACACTAAC
RP_AHA2	ATCACCACCTTTGCAATGAAC
**RT PCR**	
RT-NTR1.5	F: GGTCGCTGCAACGAAGAAATC<br>R: CAAAGCGACGCTAAGGATGTC
RT_AHA2	F: TGGTTGGGATGCTTGCTGAT<br>R: AGAGCAGGGGCATCATTGAC
RT_GAPC	F: GCAGCTCACTTGAAGGTTTG<br>R: GACTTCGTTGGCGACAACAGG
**Split ubiquitin**	
NRT1.5 (attB1/attB2)	F: ggggacaagtttgtacaaaaaagcaggcttgATGTCTTGCCTAGAGATTTAT<br>R: ggggaccactttgtacaagaagctgggttTTAGACTTTAGAATCCTTCTC
AHA2 (attB1/attB2)	F: ggggacaagtttgtacaaaaaagcaggcttgATGTCGAGTCTCGAAGATATCAAG<br>R: ggggaccactttgtacaagaagctgggtTCTACACAGTGTAGTGACTGGGAG
NRT1.5-Cub	F: cccaagcttGATGTCTTGCCTAGAGATTTATAACAAAGACAC<br>R: ccccaagcttATGACTTTAGAATCCTTCTCTCGTTCCTC
**BiFC2in1**	
NRT1.5 (attB1/attB4)	F: ggggacaagtttgtacaaaaaagcaggcttgATGTCTTGCCTAGAGATTTAT<br>R:gggggcaactttgtatagaaaagttgggtgTTAGACTTTAGAATCCTTCTC
AHA2 (attB2/attB3)	F: ggggacaactttgtataataaagttggtATGTCGAGTCTCGAAGATATCAAG<br>R: ggggaccactttgtacaagaaagctgggttCTACACAGTGTAGTGACTGGGAG
**Complementation of *nrt1.5***	
eGFP (PstI/PstI)	F:aaggattctaaagtcctgcagATGGTGAGCAAGGGCGAG<br>R:atacgaacgaaagctctgcagTTACTTGTACAGCTCGTCCATG
**Mutagenesis of *NRT1.5***	
NRT1.5^{G209E}	F: GTGTTCGAAAAGAGCGATTCGAGGTTTAAAGCAAGGT<br>R: GTGTTCGAAAAGAGCGATTCGAGGTTTAAAGCAAGGT
**Yeast expression**	
NRT1.5 (BamHI/HindIII)	F: cgggatccATGTCTTGCCTAGAGATTTATA<br>R: cccaagcttTTAGACTTTAGAATCCTTCTC
AHA2 (BamHI/HindIII)	F: cgggatccATGTCGAGTCTCGAAGATATCAAG<br>R: cccaagcttCTACACAGTGTAGTGACTGGGAG
NRT1.5 (PstI-SacII)	F:aaaactgcaggATGTCTTGCCTAGAGATTTATAACAAAGACAC<br>R:atccccgcggTTAGACTTTAGAATCCTTCTCTCGTTC
**Tags**	
eGFP (BamHI/HindIII)	F: cgggatccATGGTGAGCAAGGGCGAGGA<br>R: cccaagcttTTACTTGTACAGCTCGTCCA

Supplementary Table 2: Composition of fertilization solutions.

Fertilization solutions are based on the composition of modified 0.5 × Murashige-Skoog medium (0.5 × MS) and used to water unfertilized soil. All solutions were freshly prepared on the day of watering.

Fertilizer solutions	0K	LK	HK
	0 mM K^+<br>1 mM NO_3^-	1 mM K^+<br>1 mM NO_3^-	10 mM K^+<br>10 mM NO_3^-
**Macroelements**			
KNO_3	0 mM	1 mM	10 mM
KCl	0 mM	0 mM	0 mM
$Ca(NO_3)_2$	1 mM	0 mM	0 mM
NaH_2PO_4	1 mM	1 mM	1 mM
$MgSO_4$	1 mM	1 mM	1 mM
$CaCl_2$	1.5 mM	1.5 mM	1.5 mM
**Microelements**			
H_3BO_4	0.05 mM	0.05 mM	0.05 mM
$MnSO_4$	0.05 mM	0.05 mM	0.05 mM
$ZnSO_4$	15 µM	15 µM	15 µM
KI	2.5 µM	2.5 µM	2.5 µM
Na_2MoO_4	0.5 µM	0.5 µM	0.5 µM
$CuSO_4$	0.05 µM	0.05 µM	0.05 µM
$CoCl_2$	0.05 µM	0.05 µM	0.05 µM
**Iron**			
Fe-EDTA	0.05 mM	0.05 mM	0.05 mM
MES	0.5 g/l (pH 5.5)	0.5 g/l (pH 5.5)	0.5 g/l (pH 5.5)

Supplementary Table 3: Putative interactors of NRT1.5 from the split ubiquitin screen.

Protein subcellular localization predictions are from the ARAMEMNON 8.1 (http://aramemnon.uni-koeln.de; Schwacke et al., 2003) and SUBA4 (https://suba.live; Hooper et al., 2017) databases. PM, plasma membrane. VM, vacuolar membrane. ER, endoplasmic reticulum.

Gene ID	Name	Functional context	Subcellular localization
At5g16830	vesicle transport syntaxin-type t-SNARE protein (AtSYP21/AtPEP12)	Vesicle trafficking: post-Golgi trafficking	VM, Golgi, PM
At2g33120	VAMP222	Vesicle associate membrane protein 722	VM, endosome, plasmadesmata, PM
At2g39010	PIP2.6	PM intrinsic protein 2E	Plasmodesmata, PM, VM
At3g56240	CCH	Copper chaperone	Apoplast, chloroplast
At4g30190	AHA2	PM- H^+ ATPase 2	PM, VM, plasmadesmata

Supplementary Figure 1

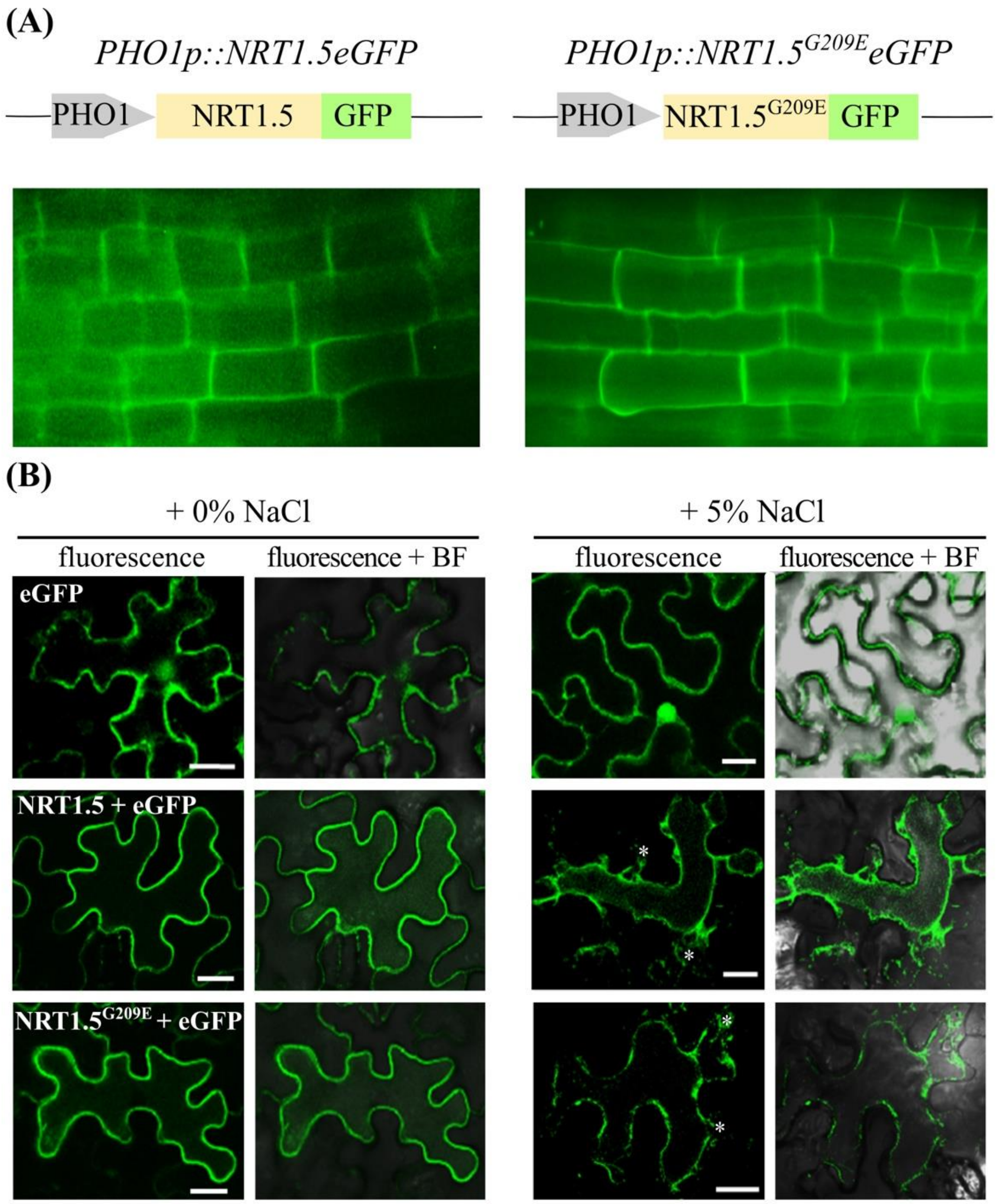

Supplementary Figure 1: Fluorescence microscopy of NRT1.5 localization. A. Epifluorescence microscopy detection of GFP signals in 10 days-old transgenic *Arabidopsis thaliana* *PHO1p::NRT1.5eGFP* and *PHO1p::NRT1.5^{G209E}eGFP* seedlings grewn in 0.5 x MS medium (pH 5.5). **B.** Cellular localization of eGFP, NRT1.5eGFP and NRT1.5^{G209E}eGFP in *Nicotiana benthamiana* epidermis cells two days after agroinfiltration was analyzed by confocal laser scanning microscopy ('fluorescence'). Cellular plasmolysis was induced by incubation in 5% NaCl during microscopic visualization. Asterisks indicate areas between plasmolyzed cells and where Hechtian strands are noticeable. The left panels shown the eGFP (green) signal and the right panels show the eGFP signal overlayed with the bright field picture ('fluorescence + BF'). The scale bar represents 20 µm.

Supplementary Figure 2

(A)

Normalized expression relative to *AHA2* in Col-0 seedlings

treatment	gene / genotype	*AHA1*	*AHA2*	*AHA3*	*AHA4*	*AHA5*	*AHA6*	*AHA7*	*AHA8*	*AHA9*	*AHA10*	*AHA11*
HK	Col-0	0.51	1.00	0.10	$6x10^{-3}$	0.01	$6x10^{-4}$	0.01	$3x10^{-4}$	$1x10^{-4}$	$1.6x10^{-3}$	$5x10^{-3}$
	nrt1.5	0.32	0.47	0.10	$1x10^{-3}$	0.01	$1x10^{-3}$	0.02	$7x10^{-4}$	$1.3x10^{-4}$	$1.1x10^{-3}$	$6x10^{-3}$
	aha2	0.45	0.03	0.08	$6x10^{-3}$	0.01	$1x10^{-4}$	0.02	$1x10^{-3}$	$0.4x10^{-4}$	$1.3x10^{-3}$	$7x10^{-3}$
	nrt1.5/aha2	0.57	0.04	0.09	$7x10^{-3}$	0.01	$6x10^{-4}$	0.03	$1.2x10^{-3}$	$1x10^{-4}$	$1.1x10^{-3}$	$15x10^{-3}$
LK	Col-0	0.60	1.00	0.10	0.03	$5x10^{-3}$	$4.5x10^{-3}$	0.02	$2x10^{-4}$	$3x10^{-5}$	$1.4x10^{-4}$	$1x10^{-3}$
	nrt1.5	0.67	1.01	0.07	0.02	$10x10^{-3}$	$3x10^{-4}$	0.01	$7x10^{-4}$	$1.7x10^{-4}$	$9x10^{-4}$	$7x10^{-3}$
	aha2	0.65	0.01	0.07	0.02	$12x10^{-3}$	$1.2x10^{-3}$	0.02	$8x10^{-4}$	$2.5x10^{-4}$	$13x10^{-4}$	$1x10^{-3}$
	nrt1.5/aha2	0.80	0.03	0.08	0.02	$12x10^{-3}$	$5x10^{-4}$	0.02	$8x10^{-4}$	$1.8x10^{-4}$	$8x10^{-3}$	$6x10^{-3}$

(B)

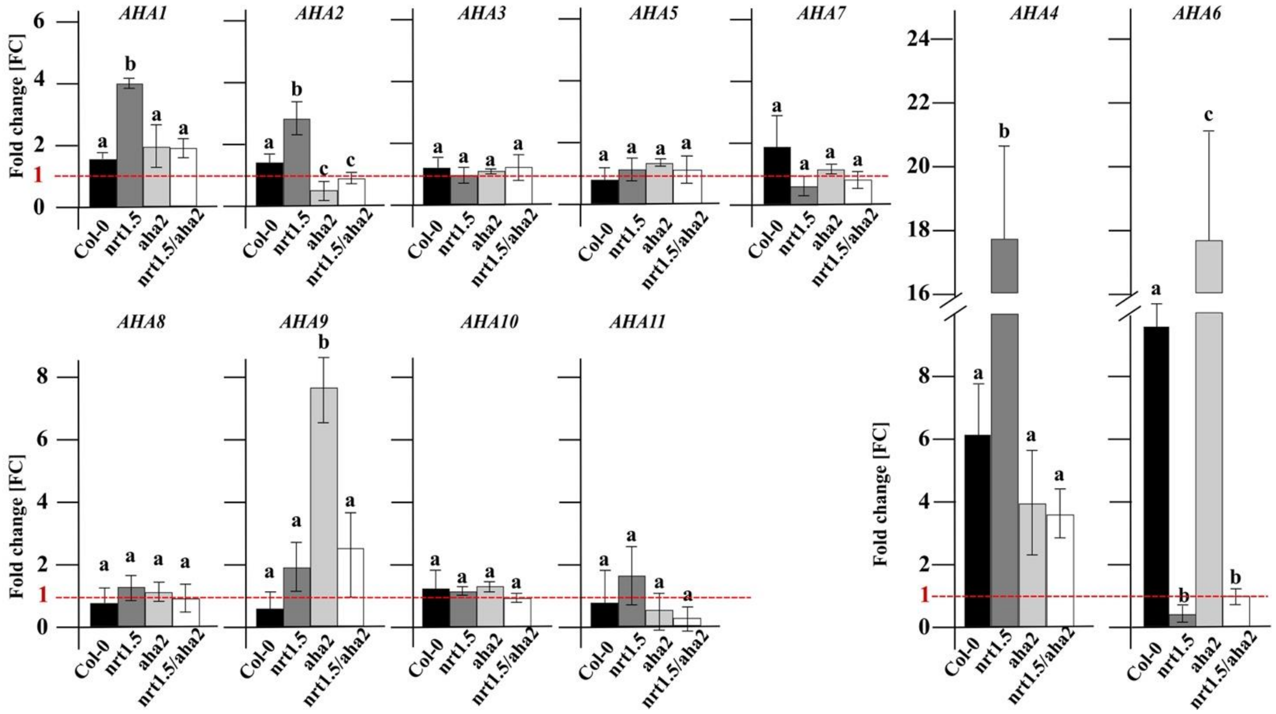

Supplementary Figure 2: Relative expression levels of the 11 H^+-ATPase family members. A. Expression levels of the 11 H^+-ATPase family members (*AHA1–AHA11*) in Col-0, *nrt1.5*, *aha2*, and *nrt1.5/aha2* seedlings grown in HK or LK conditions relative to the expression of *AHA2* in Col-0 (yellow shading). **B.** Gene expression changes of the 11 H^+-ATPase family members (*AHA1–AHA11*) in Col-0, *nrt1.5*, *aha2*, and *nrt1.5/aha2* seedlings under LK supply. Seedlings grew for five days in 0.5 x MS media, then half of them were transferred to LK medium and the other half to 0.5 x MS. Fourteen days later all seedlings were harvested. Shown is the fold change of expression (FC) of each genotype in LK relative to the expression in 0.5 x MS medium. The dotted red lines indicate the relative expression (=1) in 0.5 x MS. Different letters indicate statistically significant differences (Tukey's test) between mutants and Col-0 with $P < 0.05$, (means ± SD, n =3).

Supplementary Figure 3

(A)

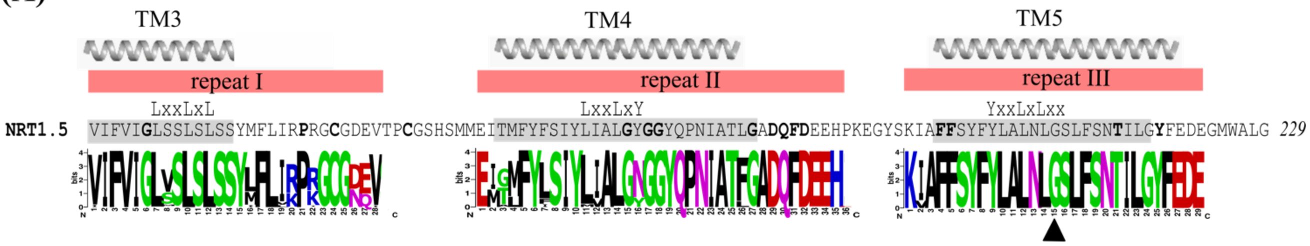

(B)

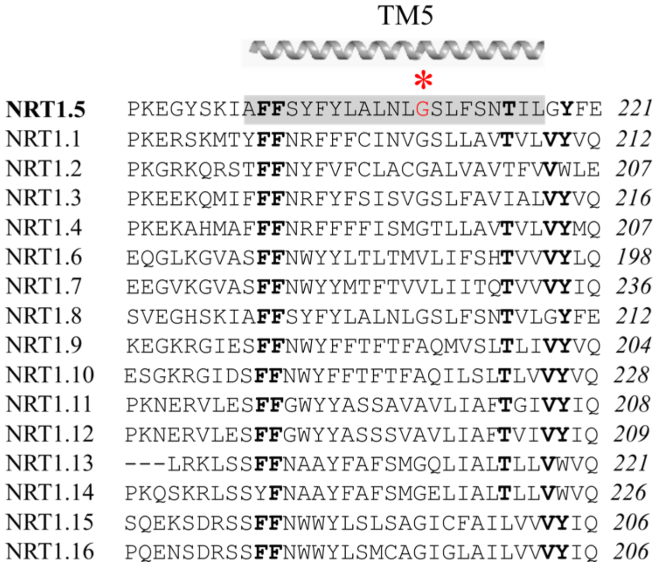

Supplementary Figure 3: Sequence motif repeats in NRT1.5. A. Sequence motif repeats in NRT1.5. Predicted helical NRT1.5 regions are shown on top. The red bars below indicate the sequence motif repeats I, II and III found in NRT1.5 by RADAR (www.ebi.ac.uk/Tools/pfa/radar/) and LRRfinder (www.lrrfinder.com). They encompass the transmembrane spans TM3, TM4 and TM5 (highlighted in grey) that contain putative leucin-rich repeats. Mutation G209E in TM5 is marked with a black arrowhead. Below the NRT1.5 protein sequence, the sequence logos of repeats I, II, and III generated from BLASTP alignments of the 100 NRT1.5 orthologs with the highest BLASTP scores from different plant species are shown. The sequence logos were generated with WebLogo (http://weblogo.berkeley.edu/logo.cgi). **B.** Protein sequence alignment of AtNRT1.5 TM5 with all NRT1 family members from *Arabidopsis thaliana*. The model-based sequence alignment of AtNRT1.5 amino acids 177 to 221, encompassing TM5, with all AtNRT1 family members was generated with Promals3d (http://prodata.swmed.edu/promals3d/promals3d.php). Conserved amino acid residues are shown in bold letters (≥ 80% conservation). The position of Gly^{209} in NRT1.5 and the respective position in the the other Arabidopsis NRT1 proteins is indicated in a red box.

Arvidsson, S., Kwasniewski, M., Riaño-Pachón, D.M., and Mueller-Roeber, B. (2008). QuantPrime--a flexible tool for reliable high-throughput primer design for quantitative PCR. *BMC Bioinformatics* 9**,** 465. doi: 10.1186/1471-2105-9-465

Hooper, C.M., Castleden, I.R., Tanz, S.K., Aryamanesh, N., and Millar, A.H. (2017). SUBA4: the interactive data analysis centre for Arabidopsis subcellular protein locations. *Nucleic Acids Res* 45**,** D1064-D1074. doi: 10.1093/nar/gkw1041

Schwacke, R., Schneider, A., Van Der Graaff, E., Fischer, K., Catoni, E., Desimone, M., Frommer, W.B., Flügge, U.I., and Kunze, R. (2003). ARAMEMNON, a novel database for Arabidopsis integral membrane proteins. *Plant Physiol* 131**,** 16-26. doi: 10.1104/pp.011577

qPCR raw data for Supplementary Figure 2

qPCR assay	Gene	Genotype	Cq HK	Cq LK
1	UBQ10	Col-0	16,4962793	18,6652784
2	UBQ10	Col-0	17,3271764	15,6489274
3	UBQ10	Col-0	16,6513212	16,813487
1	UBQ10	nrt1.5	17,5412648	15,6761893
2	UBQ10	nrt1.5	15,8318459	17,0540527
3	UBQ10	nrt1.5	16,9298512	15,3448764
1	UBQ10	aha2	15,1552987	16,0259487
2	UBQ10	aha2	16,2458374	16,9577476
3	UBQ10	aha	15,7376628	15,5065568
1	UBQ10	nrt1.5/aha2	16,8391379	15,5154521
2	UBQ10	nrt1.5/aha2	16,3247338	15,7095145
3	UBQ10	nrt1.5/aha2	15,4772989	15,9276706
1	AHA1	Col-0	18,0193045	18,4807163
2	AHA1	Col-0	18,5219351	18,2132349
3	AHA1	Col-0	19,7127832	18,3227421
1	AHA1	nrt1.5	20,7864393	16,6163406
2	AHA1	nrt1.5	17,2058025	18,2645373
3	AHA1	nrt1.5	20,1349571	16,6575705
1	AHA1	aha2	16,6306992	17,6408083
2	AHA1	aha2	18,3157958	17,6370299
3	AHA1	aha	18,5007567	16,7876068
1	AHA1	nrt1.5/aha2	19,2043978	16,3547321
2	AHA1	nrt1.5/aha2	18,0148993	16,66493
3	AHA1	nrt1.5/aha2	16,7102781	16,8002697
1	AHA2	Col-0	17,4062943	17,8101735
2	AHA2	Col-0	17,9721821	17,6072193
3	AHA2	Col-0	17,9583157	17,4082033
1	AHA2	nrt1.5	19,833144	16,7633753
2	AHA2	nrt1.5	16,8283292	16,7247965
3	AHA2	nrt1.5	19,7909059	16,2757001
1	AHA2	aha2	20,156857	22,3976895
2	AHA2	aha2	21,6701573	24,3785866
3	AHA2	aha	22,905759	22,9848819
1	AHA2	nrt1.5/aha2	21,013038	21,1444981
2	AHA2	nrt1.5/aha2	22,8252861	21,6662623
3	AHA2	nrt1.5/aha2	21,2743446	21,3365758
1	AHA3	Col-0	21,0863828	21,2317624
2	AHA3	Col-0	20,4947069	20,6946323
3	AHA3	Col-0	21,5527600	21,0148689
1	AHA3	nrt1.5	21,5814790	20,0075257
2	AHA3	nrt1.5	19,5546203	20,7596543
3	AHA3	nrt1.5	21,9774340	20,5159236
1	AHA3	aha2	19,4865248	20,5167459
2	AHA3	aha2	20,7102566	20,5387745
3	AHA3	aha	20,5233056	20,4668714
1	AHA3	nrt1.5/aha2	21,5673971	19,4358459
2	AHA3	nrt1.5/aha2	20,5374477	19,9329458
3	AHA3	nrt1.5/aha2	19,8796360	20,4567768
1	AHA4	Col-0	23,0004553	22,4703193
2	AHA4	Col-0	25,3731349	22,4660531
3	AHA4	Col-0	26,7809589	23,1880191
1	AHA4	nrt1.5	28,1112704	22,2266954
2	AHA4	nrt1.5	26,5823913	22,5930498
3	AHA4	nrt1.5	26,9198017	22,134888
1	AHA4	aha2	23,0548361	22,8885363
2	AHA4	aha2	24,6177063	22,922335
3	AHA4	aha	24,4028788	21,9426387
1	AHA4	nrt1.5/aha2	25,9168201	21,9871917
2	AHA4	nrt1.5/aha2	24,7084150	21,6375859
3	AHA4	nrt1.5/aha2	22,3213029	22,3893675
1	AHA5	Col-0	24,7932085	25,9440229
2	AHA5	Col-0	24,6794654	25,5256494
3	AHA5	Col-0	24,5429453	24,5578579
1	AHA5	nrt1.5	25,5012724	22,749416
2	AHA5	nrt1.5	22,0821834	23,6201652
3	AHA5	nrt1.5	24,7929136	23,2554078
1	AHA5	aha2	22,4185354	23,0545423
2	AHA5	aha2	23,7783384	23,1588708
3	AHA5	aha	23,0865932	23,2525408
1	AHA5	nrt1.5/aha2	23,9780089	22,1331338
2	AHA5	nrt1.5/aha2	23,0975572	22,733312
3	AHA5	nrt1.5/aha2	22,7541350	23,2923093

qPCR assay	Gene	Genotype	Cq HK	Cq LK
1	AHA6	Col-0	28,2594441	25,4662276
2	AHA6	Col-0	28,8063511	25,0424832
3	AHA6	Col-0	28,1341620	25,6676882
1	AHA6	nrt1.5	30,8395834	27,5817571
2	AHA6	nrt1.5	26,5894922	28,3099875
3	AHA6	nrt1.5	25,8345878	29,5041601
1	AHA6	aha2	28,2092288	26,4358477
2	AHA6	aha2	30,5015586	26,6251834
3	AHA6	aha	31,4664916	26,1016448
1	AHA6	nrt1.5/aha2	25,5780094	27,1375875
2	AHA6	nrt1.5/aha2	28,4900007	27,3981642
3	AHA6	nrt1.5/aha2	25,9455993	27,606543
1	AHA7	Col-0	24,0232458	23,9374637
2	AHA7	Col-0	24,0148012	22,6587419
3	AHA7	Col-0	24,0440032	23,8584062
1	AHA7	nrt1.5	24,5424184	22,514627
2	AHA7	nrt1.5	21,7638222	22,8348422
3	AHA7	nrt1.5	22,8789913	23,7116485
1	AHA7	aha2	20,9268073	22,5183208
2	AHA7	aha2	22,9834800	22,4109378
3	AHA7	aha	22,4622757	22,2272967
1	AHA7	nrt1.5/aha2	22,6208060	21,6303468
2	AHA7	nrt1.5/aha2	22,1906047	22,0992125
3	AHA7	nrt1.5/aha2	21,9889670	22,6523219
1	AHA8	Col-0	29,2451686	30,5775551
2	AHA8	Col-0	29,8911840	29,4128125
3	AHA8	Col-0	29,5277031	30,9431137
1	AHA8	nrt1.5	29,0300104	26,7866531
2	AHA8	nrt1.5	25,2455106	26,9559432
3	AHA8	nrt1.5	30,3203546	27,7143257
1	AHA8	aha2	25,9774099	27,4952188
2	AHA8	aha2	27,1192402	26,7651062
3	AHA8	aha	26,8911415	26,6505146
1	AHA8	nrt1.5/aha2	28,1975249	25,859877
2	AHA8	nrt1.5/aha2	26,2180739	27,0208518
3	AHA8	nrt1.5/aha2	26,2987974	26,9398425
1	AHA9	Col-0	29,9978948	34,666485
2	AHA9	Col-0	30,7967266	32,0906108
3	AHA9	Col-0	32,1951821	31,0461863
1	AHA9	nrt1.5	31,7699840	28,6645215
2	AHA9	nrt1.5	28,3276141	29,9000328
3	AHA9	nrt1.5	31,9624130	28,7139082
1	AHA9	aha2	30,2309703	28,6834669
2	AHA9	aha2	30,7848025	28,5122066
3	AHA9	aha	32,5649112	28,9054604
1	AHA9	nrt1.5/aha2	31,4223497	27,938989
2	AHA9	nrt1.5/aha2	29,5342260	28,9105283
3	AHA9	nrt1.5/aha2	30,4309028	29,4307004
1	AHA10	Col-0	26,0354225	26,6059907
2	AHA10	Col-0	27,2780312	26,5963573
3	AHA10	Col-0	27,8727594	28,2174361
1	AHA10	nrt1.5	28,7574131	26,6179793
2	AHA10	nrt1.5	25,6439125	26,552388
3	AHA10	nrt1.5	28,3618511	26,847203
1	AHA10	aha2	26,1925727	26,2348247
2	AHA10	aha2	26,3058348	26,5020276
3	AHA10	aha	26,2392607	26,1878101
1	AHA10	nrt1.5/aha2	27,4225973	26,4101203
2	AHA10	nrt1.5/aha2	26,8337849	26,7855755
3	AHA10	nrt1.5/aha2	26,5543445	26,5739635
1	AHA11	Col-0	27,0439961	27,5015521
2	AHA11	Col-0	23,9954668	24,7911839
3	AHA11	Col-0	25,6547572	29,3886136
1	AHA11	nrt1.5	25,904244	22,8413008
2	AHA11	nrt1.5	22,6081015	23,8414952
3	AHA11	nrt1.5	26,6036467	24,4872312
1	AHA11	aha2	23,6546413	25,2152389
2	AHA11	aha2	24,5168552	32,7365982
3	AHA11	aha	23,0837513	24,1582232
1	AHA11	nrt1.5/aha2	22,5564127	22,7226148
2	AHA11	nrt1.5/aha2	23,3757287	24,5786506
3	AHA11	nrt1.5/aha2	23,788687	23,7044034
